# *In vitro* identification of antimicrobial hemolytic lipopeptide from halotolerant *Bacillus *by Zymogram, FTIR, and GC mass analysis

**DOI:** 10.22038/IJBMS.2021.53419.12022

**Published:** 2021-05

**Authors:** Shekoofeh Sadat Etemadzadeh, Giti Emtiazi

**Affiliations:** 1 Department of Cellular and Molecular Biology and Microbiology, Faculty of Biological Science and Technology, University of Isfahan, Isfahan, Iran

**Keywords:** Antimicrobial, Bacillus halotolerans, Hemolysin, Hydrogel, Lipopeptide, Polyacrylamide gel

## Abstract

**Objective(s):**

The multi-drug resistant bacteria and clinical infections are some of the biggest global concerns, so new drugs are needed. Antimicrobial peptides and lipopeptides are new bioactive agents with great potential that can become a new strategy for clinical applications.

**Materials and Methods:**

Some Bacillus strains were isolated based on hemolytic antimicrobial production from the soil. The extracellular proteins were extracted by acidic precipitation and chloroform/methanol method and analyzed by SDS-PAGE electrophoresis and stained with Sudan black. The black fragment was purified and characterized by FTIR, GC/MS, and HPLC analysis to demonstrate the presence of lipids and proteins. The anti-microbial ability and stability of the purified lipopeptide were assayed by the Kirby-Bauer method. Also, it was examined for metal removal.

**Results:**

A new *Bacillus halotolerans* strain SCM 034 with hemolytic antimicrobial production was isolated. According to GC/MS (detecting C16, C17) and HPLC (detecting leucine, glutamic acid, valine, arginine, glycine, and aspartic acid) data, the black fragment was lipopeptide. Polyacrylamide hydrogel containing lipopeptide and gel purified lipopeptide showed anti-microbial activities against *S. aureus* and *S. cerevisiae* that were stable for a few months. Also, the lipopeptide was useful for cation removal and decreased cobalt, nickel, and calcium by 10.81 %, 24.39 %, and 34 %, respectively.

**Conclusion:**

Production of antibacterial lipopeptide hemolysin from this strain is reported for the first time and according to the results, lipopeptides have unique properties with biomedical and pharmaceutical applications. Also, polyacrylamide hydrogel lipopeptide is a promising candidate for wound healing.

## Introduction

Today one of the biggest global concerns is the continuously increasing drug resistance in bacteria following the overuse of synthetic drugs and antibiotics, while most of them can damage the environment and microbial balance (1, 2). On the other hand, multidrug resistance has led to increasing nosocomial infections, which are major concerns of the Centers for Disease Control and Prevention (CDC) (3, 4). Nosocomial infections are especially caused by Gram-positive cocci such as *Staphylococcus* *aureus*. Several factors, including multi-layered biofilm in *S. aureus*, provide resistance to host immune defenses and antimicrobial agents (3, 5, 6). Cells with biofilm can be more resistant up to 1,000-fold to antibiotics and most antibiotics cannot overcome biofilm-forming bacterial pathogens (3, 4, 6), therefore, new anti-biofilm agents and antibiotics like lipopeptides with a new mode of action are needed (1, 2, 6, 7). 

Lipopeptides are a type of biosurfactant which are mainly divided into three groups: surfactin, iturin, and fengycin families (1, 7). Biosurfactants are biologically surface-active and amphipathic agents with a hydrophilic and a hydrophobic domain which reduce the interfacial tension between oil and water and are mostly produced by microorganisms (8, 9). Some advantages of biosurfactants compared with synthetic surfactants are low toxicity, high biodegradability, and ecological acceptability, so they could be used in different industries such as food, pharmaceuticals and cosmetics, oil recovery, and oil spill removal (8, 10). 

Lipopeptides are produced by *Bacillus *species (such as *Bacillus subtilis*, *B. licheniformis *and

*B. polymyxa *(11, 12)) as efficient microbial factories and contain a hydrophilic cyclic peptide head group (L- and D- amino acids) attached to hydrophobic long alkyl chain (C10-C17) to form a non-ribosomal cyclic structure (1, 7, 13). Lipopeptides are cationic, anionic, or neutral in nature (14) and due to their structure can act as bioactive agents to disturb the functions and structure of microbial membranes, increase the membrane permeability, modify the surface hydrophobicity, affect the development of ﬂagella (for anti-adhesive properties), and alter the biofilm dispersion process, so they can be used as antifungal, antiviral, antiamoebocytic, antimycoplasma, antiadhesive, and antitumor agents and have applications in biomedicine, pharmaceutics, food biotechnology, and agricultural industry (1, 7, 15). Other microorganisms with the ability of lipopeptide production belong to *Pseudomonas*, *Streptomyces *(16), *Aspergillus *(17), *Serratia*, and *Actinoplanes *(12).

Extremophiles are a group of organisms that adapt to extreme environmental conditions such as high/low temperature, pH, salinity, and pressure, while these conditions are difficult for most life forms to survive. Extraordinary survival strategies have made them suitable for use in biotechnology, in various fields such as environment (bioremediation, biological degradation, and biocontrol), industrial (biomining, biofuels, and food), and medicine (antibiotics, antifungals, and antitumor molecules). Despite recent advances, using extremophile metabolites is very limited, especially in medical applications (18, 19).

Due to increased pathogen multi-drug resistance, the present study aimed to screen antimicrobial agents of natural origin from new hemolytic *Bacillus* sp. and identify them by Fourier Transform Infrared, Gas Chromatography/Mass Spectrometry and High-performance liquid chromatography (HPLC) analysis.

## Materials and Methods

***Isolation of hemolytic Bacillus sp. with antimicrobial properties***

Primary screening was based on isolation of spore-forming *Bacillus* sp. bacteria. So, soil samples from different regions of Kerman, Iran were collected in sterile plastic bags (from the upper layer 0–6 cm depth), 1 g of which was added to 9 ml sterile distilled water and strongly stirred with a vortex. The soil suspensions were heated in a bain-marie at 80 °C for 10 min to remove the vegetative bacteria and select the spore-forming bacteria. 100 µl of each suspension was cultured on Nutrient Agar (N.A) medium plates and were incubated aerobically at 30 °C for 24 hr. The grown colonies of *Bacillus* sp. were purified by repeated streaking on the N.A medium (20).

Then, the hemolysis test was performed to select hemolytic *Bacillus* sp. by culturing the purified isolates on Blood Agar (B.A) medium and incubating at 37 °C for 24 hr. Also, antimicrobial tests against *S.* *aureus*, *Pseudomonas* *aeruginosa, Escherichia coli*, and *Saccharomyces* *cerevisiae* were examined by the Kirby-Bauer test for the isolated hemolytic *Bacillus* sp. (21), in such a way that 0.5 McFarland (a bacterial suspension containing 1.5 × 10^8^ CFU/ml: colony-forming units per milliliter) concentration of *S. aureus*, *P. aeruginosa, E. coli*, and *S. cerevisiae* was prepared and spread over the N.A plate with a sterile swab. Then, one colony of the isolated hemolytic *Bacillus* sp. was placed on the agar plates and incubated (at 37 °C for *S. aureus*, *P. aeruginosa, and E. coli*, and at 30 °C for *S. cerevisiae*) to form the inhibition zone (the experiment was repeated three times).

***Protein extraction from the isolated hemolytic Bacillus sp. and Polyacrylamide Gel Electrophoresis (PAGE)***

In order to produce the protein, 10 % inoculation of the overnight bacterial cultures with 0.5 McFarland concentration was added to 200 ml Nutrient Broth (N.B) medium and incubated on an orbital shaker (180 rpm: revolutions per minute) at 30 °C for 24 hr to the high amount of protein production. Afterward, the medium cultures were centrifuged at 6000 rpm for 20 min to separate the bacterial cells, and the supernatant was used for extracellular protein extraction. At first, the acidic precipitation method was performed. 6 N hydrochloric acid (HCl) was gradually added to adjust pH to 2.0. After storing the samples overnight at 4 °C, centrifuging (6000 rpm, 4 °C) was done for 10 min to collect the precipitates (21, 22). Then chloroform/methanol (2: 1, v/v) was added for further purification, shaken for 20 min, and centrifuged for 10 min (6000 rpm). Finally, the chloroform phase (the lower phase) was collected and dried overnight at room temperature (8, 21). The protein concentration, if required, was determined by the Bradford protein assay (23). 

To determine the protein profile SDS-PAGE analysis was performed. The extracted proteins were dissolved in PBS (Phosphate Buffered Saline), the concentrations of protein were measured with Bradford protein assay and then loaded on the polyacrylamide gel (12 % (w/v), separating gel, and 5 % (w/v) stacking gel). The samples were not treated with 2- mercaptoethanol (2-ME) or heat, and the voltage was about 100 v. The gel was stained by Coomassie brilliant blue R-250 and destained with methanol/ acetic acid/ water (45: 10: 45, v/v).

***Isolate identification and growth curve studies***

After primary experiments, the most appropriate hemolytic lipopeptide-producing strain with anti-microbial properties was selected and more studies were done on it. The growth rate of the selected isolate was investigated in N.B medium, during 72 hr incubation at 30 ˚C with shaking at 180 rpm by recording the absorbance at 600 nm using a spectrophotometer in 3 repeats (24). For isolate identification, morphological studying, and gram staining, biochemical and physiological confirmation tests were performed (25). For molecular identification, the isolate was cultured on an N.A medium. After incubation (18 hr at 30 ˚C), genomic DNA was extracted by the boiling method. Briefly, the colonies were suspended completely in 500 µl sterile distilled water and heated at 100 ˚C in a water bath (for 10 min) and then centrifuged at 7000 rpm for 15 min. Finally, 200 µl of the aqueous supernatant was transferred to a sterile microtube as the DNA template for the PCR (Polymerase Chain Reaction) process. Amplification of the 16S rRNA gene was performed at a volume of 50 µl by using universal primers 1492R and 27F. The PCR mixture contained 39 µl sterile distilled water, 5 µl 10x PCR buffer, 1.4 µl MgCl_2_ (50 mmol), 1 µl dNTP (10 mmol), 1 µl of each primer, 1 µl of DNA template (more than 100 ng/µl), and 0.6 µl of Taq DNA polymerase. The reaction mixture was first incubated at 94 °C for 10 min and then 35 cycles were carried out as follows: 45 sec denaturation at 94 °C, 1 min annealing at 55 °C, 1 min extension at 72 °C, and a final elongation step at 72 °C for 10 min. The resulting amplicon was analyzed on 1 % agarose gel by electrophoresis in TBE buffer. The whole 16S rRNA gene of about 1500 bp was sequenced in both directions using an automated sequencer by ABI 3730 XL (Spain).

***Determination of bacterial tolerance to salt and metals***

For determination of the growth ability of the *Bacillus* sp., the agar dilution method was used. The isolated strain was cultured on N.A medium containing 5, 10, 12, and 15 % sodium chloride (NaCl) and 1 mM of copper(II) sulfate pentahydrate (CuSO_4_.5H_2_O), zinc sulfate heptahydrate (ZnSO_4_.7H_2_O), calcium chloride dihydrate (CaCl_2_.2H_2_O), potassium dichromate(VI) (K_2_Cr_2_O_7_), manganese(II) chloride (MnCl₂), nickel(II) chloride hexahydrate (NiCl_2_.6H_2_O), cobalt(II) chloride hexahydrate (CoCl_2_.6H_2_O), cadmium nitrate tetrahydrate (Cd(NO_3_)_2_.4H_2_O), mercury(II) chloride (HgCl_2_) and lead(II) acetate Pb(C_2_H_3_O_2_)_2_, and the metal concentration was progressively increased. The bacterial growth was observed by streaking on the plates after 24–120 hr (at 30 °C) and the MIC (minimum inhibitory concentration) was determined when the isolate could not grow (the experiment was repeated three times) (26).

***Zymography for determining the effective fragment***

To determine the effective fragment in hemolysis, antimicrobial, and metal removal activity, zymography was done. For anti-microbial activity, SDS-PAGE was performed (without staining) and the gel was placed on the cultured N.A plate with *S. aureus*, *P. aeruginosa, E. coli*, and *S. cerevisiae*. For hemolysis and metal removal activity, after SDS-PAGE analysis (without staining), the gel was fixed with methanol/acetic acid/water (40: 10: 40, v/v) for 25–30 min, and washed 3 times with double-distilled water (shaking for 5 min each). For SDS removal, the gel was placed in 2.5% (v/v) Triton X-100 twice with shaking (each time for 15 min), and then washed twice with double-distilled water (each time for 20 min with shaking). The hemolysis activity was assayed by placing the gel on the B.A plate (21) and for metal removal, the gels were placed in concentrated metal solutions (cobalt (CoCl_2_.6H_2_O) and calcium (CaCl_2_.2H_2_O)). 

***Staining of the bright clear fragment by sudan black B***

Sudan black B is a specific fat stain for detection of lipids (27), so it was used for lipid detection in the bright clear fragment on the polyacrylamide gel. After SDS-PAGE analysis, the polyacrylamide gel was placed overnight in the staining solution (250 mg Sudan Black, 10 ml acetone, 7.5 ml glacial acetic acid, and 40 ml water), and then washing was performed several times by destaining solution (acetone: acetic acid: water, 20: 15: 54, v/v/v) (28).

***Extraction of the bright clear fragment from SDS-PAGE gel and studying of its properties***

Hitherto, various methods have been used to extract the protein of interest from the polyacrylamide gel, like electroelution and passive diffusion (29, 30). In this experiment, lipopeptide extraction from the polyacrylamide gel was performed using elution by diffusion in methanol- chloroform as a lipopeptide extraction solvent (unpublished data). 

In this method, instantly after SDS-PAGE analysis (without fixing or staining), the bright clear fragment was cut from the gel (the bright clear fragment was visible even without staining) and divided into very small pieces using a new sterile scalpel and placed overnight in chloroform/methanol (2: 1, v/v) solution with shaking. Then centrifuging (6000 rpm, 10 min) was done and the chloroform phase (the lower phase) was collected and dried at room temperature as purified lipopeptide. SDS-PAGE analysis was performed again to ensure the bright clear fragment purification process.

***Fourier transform infrared (FTIR) analysis***

FTIR spectroscopy (JASCO, FT/IR-6300, Japan) was performed for the initial identification of the chemical components of the purified lipopeptide.

***Gas chromatography/mass spectrometry (GC/MS) analysis***

GC/MS analysis was performed to recognize the lipid component of the purified lipopeptide. The purified lipopeptide (0.005 gr) was dissolved in 500 µl chloroform, and 1 µl of the solution injected into the GC/MS analyzer (Agilent 7890A Gas Chromatograph, capillary column DB_5_ (30 m × 0.2 mm), carrier gas was helium (at 1 ml/min). This test was done twice (0, 2 months later) for determination of the stability of the lipid component of the purified lipopeptide (21). 

***High-performance liquid chromatography (HPLC) analysis***

This analysis was selected for determination of amino acid derivatives. At first, the purified lipopeptide was hydrolyzed using boiling in 6 N HCl at 110 °C for 24 hr in sealed tubes to get free amino acid, and then amino acid identification was performed by reverse-phase HPLC at 254 nm (31).

***Cation removal by the purified lipopeptide***

In this experiment, the ability of cation removal was tested by the purified lipopeptide. For heavy metal removal, 50 µl of the purified lipopeptide in PBS (22.5 µg/ml) was added to 500 µl of 1 M cobalt and nickel solution (CoCl_2_.6H_2_O, NiCl_2_.6H_2_O) and for the control sample 50 µl PBS was added instead of the metal solution. After 1 hr, centrifugation was done (12000 rpm, 10 min), and the absorbance of the supernatant for each solution was measured by a spectrophotometer at 540 nm.

For calcium removal, 500 µl of the lipopeptide was added to 500 µl of the calcium (CaCl_2_.2H_2_O) solution (0.1 gr/ml) and for the control sample 500 µl PBS was added. After 30 min, centrifugation was done (10,000 rpm, 5 min). Then the precipitated calcium was collected, dried, and measured (experiment was repeated three times) (21).

***Antimicrobial properties and stability of the purified lipopeptide***

This experiment was performed to confirm the antimicrobial activity of the purified lipopeptide by the Kirby-Bauer test and measuring the diameter of the inhibition zone. The suspensions of *S. aureus *and *S. cerevisiae *were prepared in 0.5 McFarland concentration and cultured on the N.A plate and then 3 µl of the purified lipopeptide in PBS (22.5 µg/ml) was added to it. After incubation time (24 hr at 37 °C for *S. aureus* and 48 hr at 30 °C for *S. cerevisiae*) the inhibition zone was measured (experiment was repeated three times) (21). To check the stability of this biomaterial over time, this test was performed at regular times (0, 72 hr, 1 week, 1 month, 2 months), and throughout this time it was kept at -20 ° C.

## Discussion

The spore-forming bacteria *Bacillus* sp. was isolated from the soils by heating (80 ˚C) for 10 min in a water bath and were cultured on N.A medium plates. In the next step, the isolated colonies were tested for hemolysis by culturing on B.A plates. Also, the antimicrobial activities of the hemolytic *Bacillus* sp. isolates were investigated against *S. aureus*, *P. aeruginosa, E. coli*, and *S. cerevisiae*. According to the results obtained (Table 1), two isolated strains (A1 and L1) were selected to continue the experiments.

**Figure 1:**
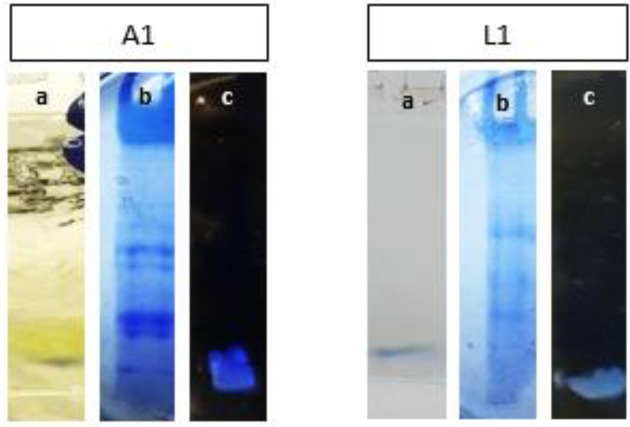
SDS-PAGE gel electrophoresis of the extracted protein from the bacterial supernatants (A1 and L1); a) Gel without staining, where the light protein fragment was observed in yellow color especially in isolated A1, b) Gel staining by Coomassie brilliant blue on a light page, c) Gel staining by Coomassie brilliant blue on a dark page, where the light protein fragment was observed as the bright clear fragment and it was sharper in isolated A1

Protein extraction from the supernatant was performed by acidic precipitation and the chloroform/methanol method, the concentrated proteins were dissolved in PBS and then analyzed by SDS-PAGE gel electrophoresis (Figure 1). A thick bright fragment (about 10 kDa) was observed on the gel in yellow color without staining for isolated A1 (Figure 1a). Gel staining was done with Coomassie brilliant blue (Figure 1b) and for observing the bright clear fragment, the gel was placed on a dark page (Figure 1c), this fragment was sharper in isolated A1. 

According to the results, isolated strain A1 was selected for the next experiments for its high antibacterial activities and higher protein production. The identification of the strain and its growth characteristics was performed. This soil bacterium was isolated from around Sarcheshmeh copper mine, Kerman, Iran, and morphological and biochemical characteristics of this spore-forming Bacillus were stated in Table 2. 

The nucleotide sequence of the amplified DNA according to the NCBI database revealed a 99.93 % homology to *B.* *halotolerans* strain DSM 8802, and the 16S rRNA gene sequence has been deposited in the Genbank as *B.* *halotolerans* strain SCM 034 (accession number MT810037).

**Figure 2:**
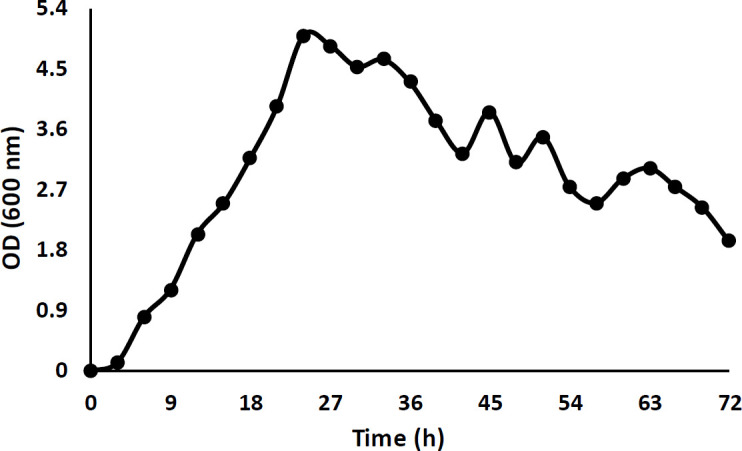
Growth curve of the isolated strain in N.B during 72 hr. The maximum growth was after 24 hr

The growth curve of this bacterium was studied for 72 hr in N.B medium (at 30 °C and 180 rpm), the maximum growth was after 24 hr (results shown in Figure 2) and the protein extraction was performed at this time. 

**Figure 3.**
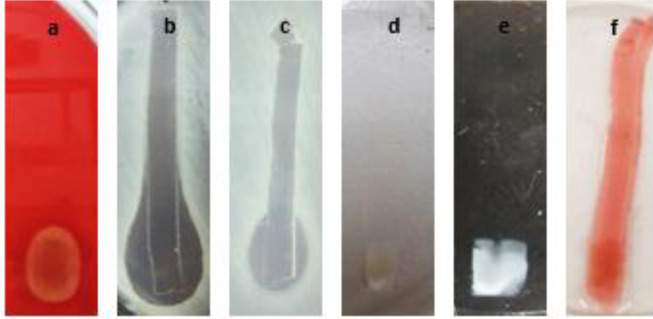
Zymography of the SDS-PAGE gel of isolated A1 in different media to determine the effective fragment; a) Hemolysis activity of the bright clear fragment on the B.A plate, b) Anti-*Staphylococcus aureus* effect of the bright clear fragment, c) Anti- *Saccharomyces cerevisiae* effect of the bright clear fragment, d) Role of bright clear fragment in calcium absorption on a light page, e) Role of bright clear fragment in the calcium absorption on a dark page, f) Cobalt adsorption activity of the bright clear fragment

The growth ability of the isolated bacterium was examined against some metals and salt. The growth was inhibited at 7 mM Pb, 7 mM Ca, 5 mM Cr, 3 mM Ni, Mn, and Cu. This strain showed very low resistance to mercury (grew so weak in only 1 mM) and it could not grow on cadmium, cobalt, and zinc. Also, the isolate could tolerate more than 12% of NaCl. Afterward, the role of extracted protein in metal adsorption was investigated by zymography in the cobalt and calcium solution, and as Figures 3d, e, and f show, only the bright clear fragment in the gel has changed and absorbed metals. 

Also, zymography indicated the bright clear fragment as the effective fragment in hemolysis and antimicrobial activity against *S. aureus* and *S. cerevisiae *(Figures 3a, b, and c), although the anti-*P. aeruginosa* and* E. coli* effects were not specified on the zymogram.

**Figure 4:**
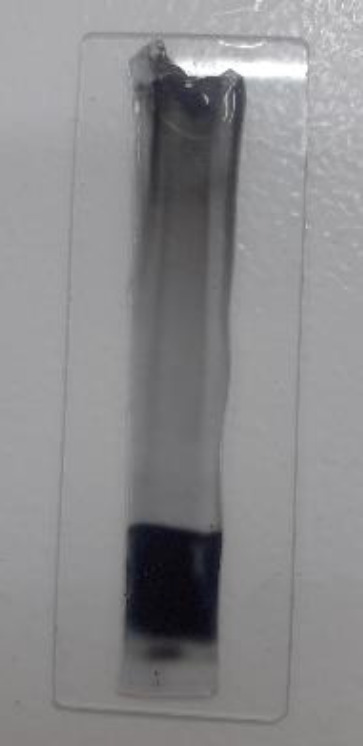
SDS-PAGE gel staining with Sudan Black B where the bright clear fragment was observed in black color because of the presence of lipid

For the initial identification of the components of the bright clear fragment, gel staining was performed with Sudan Black B and black color observation confirmed the presence of lipid (Figure 4). Therefore, the bright clear fragment with hemolytic and antimicrobial properties was determined as a lipopeptide compound which has also been reported in* B.* *halotolerans* whole genome.

In the next step, lipopeptide extraction was performed by a kind of diffusion method in methanol- chloroform; the bright clear fragment of the gel was cut and divided into very small pieces and after overnight shaking, the chloroform phase was separated and dried as purified lipopeptide.

**Figure 5:**
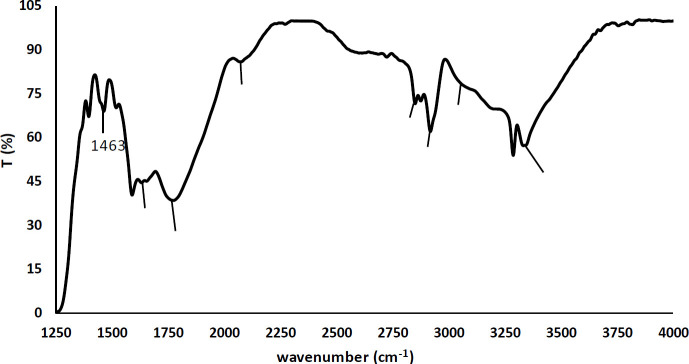
Fourier transform infrared (FTIR) spectrum of the purified lipopeptide illustrating the functional groups of lipid and peptide

The results of FTIR analysis demonstrated the presence of lipid and peptide functional groups in the purified lipopeptide (Figure 5). The results showed C=O stretching of COO^-^ from protein at 1396 cm^-1^ (32), amide II at 1513 cm^-1^ (33), ring C-C stretch of phenyl, and C=C stretching of pyridone peaks at 1589 cm^-1^ and 1628 cm^-1^, respectively (34, 35), N=C=S (isothiocyanate) group at 2072 cm^-1^ (36) and the terminal -NH_2_ and N-H stretching at 3190 cm^-1^ and 3284 cm^-1^ (2, 37), respectively. Also, peaks at 1463 cm^-1^, 2851 cm^-1^, 2919 cm^-1^, and 3064 cm^-1^ were due to C-H and CH_2_ (21, 38), 1773 cm^-1 ^and 3333 cm^-1^ were due to strong C=O (39) and O-H stretching band (40).

**Figure 6:**
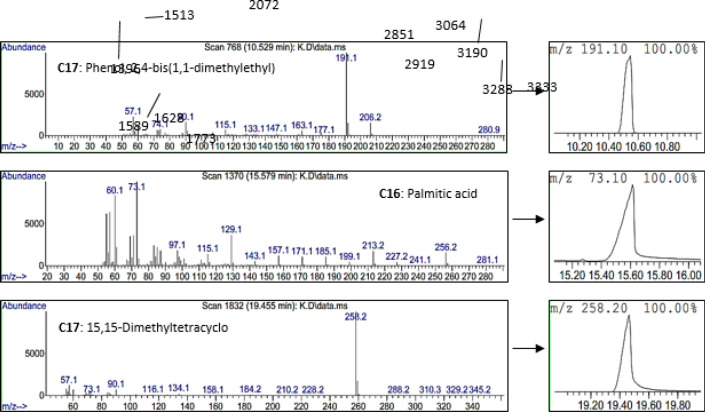
Major lipid compounds of the purified lipopeptide by GC/MS analysis: C16 and C17 which were found in bacterial lipopeptides

The purified lipopeptide was analyzed by GC/MS for recognizing the lipid components and its stability after 2 months,  generally, no differences were observed and the lipid component was stable. Also, C16 and C17 were the major compounds found in bacterial lipopeptides (like Iturin, Fengycin, and Surfactin) (Figure 6).  

HPLC analysis determined the amino acid derivatives (Table 3); leucine (43.18%), glutamic acid (9.82%), valine (9.46%), arginine (9.34%), glycine (7.71%), and aspartic acid (6.53%) had the maximum percentage in the purified lipopeptide. 

Metal removal with the purified lipopeptide using spectroscopy was 10.81 % and 24.39% for cobalt and nickel, respectively, and the amount of precipitated calcium was about 34%.

Also, the antimicrobial activity of the purified lipopeptide and its stability was examined by the Kirby-Bauer method at definite times (0, 72 hr, 1 week, 1 month, 2 months). The results (Table 4) showed, the purified lipopeptide preserved the antimicrobial properties against *S. aureus* and *S. cerevisiae* and was relatively stable for 2 months.

**Table 1:** Properties of *Bacillus* sp. isolated from the soil samples

Name	Hemolysis	Anti *S. aureus*	Anti *P. aeruginosa*	Anti *E. coli*	Anti *S. cerevisiae*
1a	+ (β)	+	+	+	+
L1	+ (β)	+	-	-	+
N1	+ (α)	+	+	-	-
N3	-	+	-	-	-

**Table 2:** Morphology and biochemical characteristics of the isolated strain from soil

Test	Result
Gram stain	Positive
Motility	Positive
Catalase activity	Positive
Oxidase activity	Positive
MR reaction	Negative
VP reaction	Positive
Citrate utilization	Negative
Urease production	Negative
ONPG	Positive

N.B: Nutrient Broth

**Table 3:** Amino acids percentage in the purified lipopeptide by High-performance liquid chromatography (HPLC) analysis

Amino acid	Percentage (%)	Amino acid	Percentage (%)
Leucine	43.18	Threonine	0.51
Valine	9.46	Alanine	2.26
Aspartic acid	6.53	Tyrosine	2.18
Glutamic acid	9.82	Phenylalanine	1.02
Arginine	9.34	Isoleucine	1.84
Glycine	7.71	Ornithine	1.72
Serine	1.99	Lysine	1.96
Glutamine	0.31	Taurine	0.12
Asparagine	0.05		

**Table 4:** Antimicrobial properties of the purified lipopeptide against *Staphylococcus aureus *and *Saccharomyces cerevisiae* during a definite time

Time	*S. aureus*	*S. cerevisiae*
0	8 mm	7 mm
72 h	8 mm	7 mm
1 week	7 mm	6 mm
1 month	6 mm	6 mm
2 months	5 mm	5 mm

## Discussion

One of the major concerns of the CDC is the increase in multi-drug resistance and clinical infections, which can be attributed to the overuse of antibiotics and microbial virulence factors like biofilms. Therefore, there is a need to find out some new alternative antimicrobial agents (2, 3, 7). Lipopeptides are a type of biosurfactant with great potential that can be used as a new method for clinical applications as antifungal, antiviral, antiameobocytic, antimycoplasma, and antitumor agents (2, 7, 15). lipopeptides have been reported to be produced by some members of *Bacillus*, *Pseudomonas*, and *Actinomycete*, but *Bacillus * strains are considered as significant microbial factories for the large-scale production of different types of lipopeptide (Surfactin, Iturin, and Fengycin) (1, 31). So, the present study aimed to screen new antimicrobial agents of natural origin like lipopeptides from hemolytic *Bacillus* sp., because due to their structure (amphipathic structure containing a hydrophilic cyclic peptide and a hydrophobic fatty acid chain), they have the ability to lyse blood cells (7, 31). After primary tests including hemolysis test, antimicrobial tests, demonstration of protein production, and comparing their production, one isolated strain (A1) was selected and identified as *B.* *halotolerans* strain SCM 034 (accession number MT810037) by biochemical and molecular methods. This isolate had anti-microbial effects against *S. aureus*, *P. aeruginosa, E. coli*, and *S. cerevisiae*, while the lipopeptide clear fragment in the zymogram had just anti-*P. aeruginosa* and* E. coli* effects which can be used as a polyacrylamide-based hydrogel for wound healing and it has advantages over other hydrogels, for example, natural collagen has low flexibility and easy degradation (41). As previously mentioned, many studies have shown the antimicrobial (antifungal, antibacterial, antiviral, and anti-biofilm forming) effects of lipopeptides from *Bacillus *strains (*B. subtilis*, *B. velezensis*, *B. mojavensis*, *B. licheniformis*, *B. amyloliquefaciens*, etc.) (2, 7, 9, 31, 42). On the other hand, the antifungal, antibacterial, and anti-nematode effects of the secondary metabolites of *B. halotolerans* have been demonstrated and introduced as a sustainable biocontrol strain (25, 43, 44). The presence of lipopeptides has been reported in* B.* *halotolerans* whole genome in NCBI. It should be noted that the old name of this bacterium was *Brevibacterium* *halotolerans* and it has been reclassified to* Bacillus* *halotolerans* (43, 45). 

The MIC of metal for *B. halotolerans *was 7 mM to Pb, 7 mM to Ca, 5 mM to Cr, 3 mM to Ni, Mn, and Cu and it grew very weak in 1 mM Hg but it had no growth on 1 mM cadmium, cobalt, and zinc. Also, it could tolerate more than 12% of NaCl. Slama *et al*. (46) studied the resistance of four *B. halotolerans *strains to lead, cadmium, cobalt, and mercury and found that all isolates resisted up to 1000 ppm lead and 100 ppm cobalt, but only one strain could grow at 50 ppm mercury and two isolates could resist 100 ppm cadmium. Also, they could tolerate up to 1200 mM NaCl. In this experiment, the role of lipopeptide in cation absorbance was shown by zymography in the cobalt and calcium solution and the heavy metal removal was proven by spectroscopy. The elimination of Co (II) and Ni (II) was 10.81 % and 24.39 %, respectively. Also, the removal of Ca^++^ was 34 % and so it can help in controlling and treating cancer and infection by reducing calcium levels (47).

The lipopeptide fragment was seen as the bright clear fragment on the SDS-PAGE gel and the lipid component was demonstrated with Sudan Black B staining. The lipopeptide fragment was extracted from the SDS-PAGE gel and its components were identified at first with FTIR, and then GC/MS and HPLC analysis. FTIR analysis indicated the functional groups of lipid and peptide, therefore, it was confirmed that the extracted substance was lipopeptide.

In GC/MS analysis, C16 and C17 lipids were identified which are found in lipopeptides from *Bacillus.* Previous studies reported that lipopeptides have different variants, and the pattern of amino acids and fatty acids depends on the producer bacterial strain and culture conditions. Lipid components can vary from C-14 to C-17 in iturin A, C-14 to C-17 in fengycin, and C-12 to C-16 or C-17 in surfactin (1, 48, 49). C16: palmitic acid which was detected in GC/MS analysis, is known to have antibacterial, antifungal, and anti-inflammatory properties (50), and C17: Phenol, 2,4-bis(1,1-dimethylethyl) has been demonstrated to have anti-biofilm formation effects mediated quorum sensing inhibiting (4). 

HPLC analysis showed the maximum percentage of amino acids is related to leucine (43.18 %), glutamic acid (9.82 %), valine (9.46 %), arginine (9.34 %), glycine (7.71 %), and aspartic acid (6.53 %). The results indicate the presence of iturin (tyrosine, asparagine, proline, serine, and glutamine), fengycin (tyrosine, threonine, glutamic acid, alanine, proline, glutamine, tyrosine, and isoleucine) and especially surfactin (leucine, valine, aspartic acid, and glutamic acid) lipopeptides of *Bacillus * strains (1, 49, 51). Fanaei and Emtiazi (31) illustrated the dominant amino acids in lipopeptide of *B. mojavensis* were negatively charged (glycine, glutamic acid, and aspartic acid), and Paduszynska *et al*. (52) demonstrated the positive charge amino acids, arginine and lysine, have a strong anti-biofilm potential.

Finally, in this experiment, the antimicrobial activity of the purified lipopeptide and its stability were studied. The purified lipopeptide which was used in very small amounts preserved its antimicrobial activity and showed remarkable anti-*S. aureus* and *S. cerevisiae* effects. These effects were stable during 72 hr, and even after 2 months 62.5 % of anti-*S. aureus* and 71.43 % of anti-*S. cerevisiae* effects were retained.

## Conclusion

In this study, a new *B.* *halotolerans *with the ability of hemolytic lipopeptide production was isolated. The lipopeptide after purifying indicated remarkable anti-microbial activities against *S. aureus* and *S. cerevisiae *with high stability even after 2 months and cation removal properties (cobalt, nickel, and calcium). According to the data from FTIR, GC/MS, and HPLC analysis, the purified lipopeptide possibly contains iturin, fengycin, and specially surfactin. So based on the obtained results, 

it is suggested that lipopeptides have a great potential to be used in different industries such as

biomedical (as antimicrobial and anti-biofilm agents or as a polyacrylamide hydrogel for wound healing) and bioremediation.
